# HER2 Aberrations in Non-Small Cell Lung Cancer: From Pathophysiology to Targeted Therapy

**DOI:** 10.3390/ph14121300

**Published:** 2021-12-14

**Authors:** Ioannis A. Vathiotis, Andriani Charpidou, Niki Gavrielatou, Konstantinos N. Syrigos

**Affiliations:** 1 Section of Medical Oncology, Third Department of Internal Medicine, National and Kapodistrian University of Athens, 11527 Athens, Greece; dcharpidou@yahoo.gr (A.C.); ksyrigos@med.uoa.gr (K.N.S.); 2 Department of Pathology, Yale School of Medicine, New Haven, CT 06510, USA; niki.gavrielatou@yale.edu

**Keywords:** NSCLC, HER2, ERBB2, trastuzumab

## Abstract

While human epidermal growth factor receptor 2 (HER2) aberrations have long been described in patients with non-small cell lung cancer (NSCLC), they have only recently been effectively targeted. Unlike patients with breast cancer, NSCLC patients can harbor either *HER2*-activating mutations or *HER2* amplification coupled with protein overexpression. The latter has also been the case for patients with acquired resistance to epidermal growth factor receptor (EGFR) tyrosine kinase inhibitors (TKIs). As preclinical data continue to accumulate, clinical trials evaluating novel agents that target HER2 have produced promising preliminary results. Here, we review existing data on HER2 aberrations in NSCLC. Starting from HER2 biology in normal and disease processes, we summarize discrepancies in HER2 diagnostic assays between breast cancer and NSCLC. Finally, to dissect the therapeutic implications of *HER2*-activating mutations versus gene amplification and/or protein overexpression, we present data from prospective clinical trials that have employed distinct classes of agents to target HER2 in patients with NSCLC.

## 1. Introduction

First identified in the 1980s as the product of *neu* oncogene in rodents by Robert Weinberg’s group, human epidermal growth factor receptor 2 (HER2; also known as ErbB2 or CD340) is one of the four members of the ErbB family of receptor tyrosine kinases (RTKs), along with ErbB1 (epidermal growth factor receptor (EGFR), HER1), ErbB3 (HER3), and ErbB4 (HER4) [1]. The initial discovery of the *ErbB2* gene in a rat neuro/glioblastoma model in 1984 was soon followed by the uncovering of its implication in breast cancer pathophysiology and prognosis, laying the groundwork for novel directions in breast cancer treatment and commencing the era of targeted therapy in modern oncology [2,3].

HER2 activation has been shown to drive oncogenic downstream signaling, promoting tumor cell proliferation and survival [4]. Consequently, HER2 targeting has been extensively investigated as a potential therapeutic strategy, demonstrating efficacy across a multitude of solid tumors. Identified in 15–20% of all breast cancers, HER2 protein overexpression and/or gene amplification has been shown to characterize an aggressive disease subgroup with high invasive and metastatic potential, resistance to hormonal and chemotherapy regimens, and poor outcome [5,6]. In 1998, the first FDA approval of trastuzumab, a monoclonal antibody (mAb) against HER2, for the treatment of metastatic breast cancer marked the beginning of the upturn of what had been a dismal natural course of HER2-positive disease [7,8]. Since then, several HER2-targeting agents, including mAbs, tyrosine kinase inhibitors (TKIs), signal transduction inhibitors, and lately, antibody–drug conjugates (ADCs) have shown preclinical and/or clinical efficacy, spanning all disease stages and treatment settings of HER2-positive breast cancer. Accordingly, gastric and gastroesophageal junction tumors, which demonstrate HER2 positivity in approximately 20% of the cases, became the second malignancy for which trastuzumab was added to standard of care, first-line chemotherapy regimens [9]. Additionally, HER2 overexpression and/or gene amplification of diverse degree has also been described in several other solid tumors including biliary tract, colon, bladder, ovarian, endometrial, head and neck and non-small cell lung cancer (NSCLC) [10]. However, targeting HER2 aberrations with conventional anti-HER2 agents has failed to replicate their breast cancer efficacy, indicating the extent of biological diversity conferred by alternative HER2 aberrations, which prevail in distinct malignancies [11,12].

Springing from the recent Food and Drug Administration (FDA) therapy designations of two agents targeting HER2, we review available data on HER2 aberrations in NSCLC. Based on the biology of this pathway in normal and disease processes, we sought to describe discrepancies in HER2 diagnostic assays that could potentially explain discordances in response to distinct classes of agents targeting HER2 in patients with NSCLC.

## 2. Biology

*Physiology.* All four ErbB receptors constitute type I transmembrane growth factor RTKs with high structural homology. They consist of an extracellular N-terminal region, which acts as their ligand-binding site, a transmembrane region, and an intracellular region, which is composed of a juxtamembrane, a kinase catalytic, and a carboxy-terminal domain [13,14]. Under physiologic conditions, ligand binding results in either homodimerization or heterodimerization, which is the required initial step for activation, and it sequentially triggers the transphosphorylation of intracellular tyrosine residues and stimulates multiple downstream signaling pathways related to cell growth, differentiation, survival, and invasion [15]. Several molecules have been identified as soluble ligands with specific binding capacity to one or more ErbB receptors; ligand–receptor specificity has been implicated in the elicitation of distinct signaling pathways, which is an effect linked to variable dimer formation and tyrosine residue phosphorylation [16]. In contrast with the other ErbB family members, HER2 is characterized as a ligand-independent receptor, as no molecule has been described to bind to its extracellular domain, which may retain an active conformation, irrespective of the presence of ligand [17,18]. Interestingly, HER2, which has the highest tyrosine kinase activity, represents the preferred partner for heterodimerization with any ErbB family member, while HER2 pairing with HER3, which in turn lacks tyrosine kinase activity completely, displays the highest signaling potency, suggesting a complementary interaction of HER2 and HER3 [19,20].

*Pathophysiology.* HER2 protein overexpression, which occurs under unknown biological mechanisms, and/or *HER2* gene amplification or transcriptional dysregulation results in up to 100-fold increase in cell-surface HER2 and consequently drives HER2-mediated tumorigenesis [21]. The increased presence of HER2 on the cell surface results in an increased formation of HER2-containing heterodimers, which is a process that has been shown to alter cell polarity and adhesion and lead to the activation of several oncogenic signaling pathways including MAPK, PI3K/Akt, phospholipase-Cγ, protein kinase C, and the Janus kinase (Jak-STAT) [22]. Although somatic mutations in the extracellular or transmembrane domain of the *neu* gene (the rodent analogue of *HER2*) have been reported in preclinical models, human HER2-positive breast tumors consistently demonstrate overexpression of wild-type HER2 [23].

**Figure 1:**
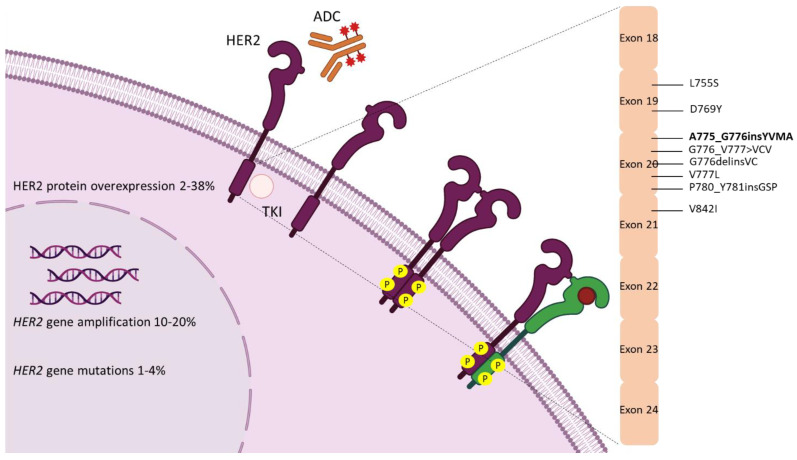
HER2 aberrations in non-small cell lung cancer. ADC, antibody–drug conjugate; TKI, tyrosine kinase inhibitor.

While breast cancer pathogenesis predominantly depends on HER2 overexpression caused by gene amplification, HER2-associated lung tumors may exhibit either HER2 overexpression or somatic mutations [24,25]. The absence of amplification and mutation overlap in NSCLC has been proposed as an explanation for the observed poor outcomes of classic anti-HER2 agents employed in breast cancer therapeutics [26,27]. The breach between the biological impact of *HER2* amplification versus mutation is further highlighted by the observation that the former has been associated with improved response to EGFR inhibition in NSCLC, whereas the latter has been linked to in vitro resistance to EGFR tyrosine kinase inhibitors (TKIs) [28,29]. Moreover, in contrast with *HER2* mutations, *HER2* amplification has been identified as a mechanism of acquired resistance to EGFR TKIs [30]. The majority of *HER2* mutations in NSCLC comprise insertions or missense mutations located in exons 18–20 and similarly to mutations in *EGFR*, which primarily involve exons 18–21, they affect the αC-helix and loop part of the kinase domain (Figure 1) [31,32]. The most commonly described *HER2* mutations in NSCLC are in-frame insertions in exon 20, which can act as proliferative drivers through constitutive activation of the receptor and downstream induction of PI3K/Akt and MEK oncogenic pathways [33]. The in-frame YVMA insertion at residue 776 of exon 20 represents the most frequently detected *HER2* variant (80–90% of all *HER2* mutations) and has been associated with more potent catalytic kinase activity and increased transforming and anti-apoptotic potential in comparison with the wild-type *HER2* gene [25,34,35]. Notably, YVMA insertion has been identified as an early event lung adenocarcinoma tumorigenesis [36]. Apart from kinase domain alterations, a germline (G660D) and a somatic (V659E) mutation of the transmembrane domain have also been identified as lung adenocarcinoma drivers through the activation of Akt signaling [37]. Extracellular domain mutations have also been described in NSCLC, and specifically, G309E and S310F have demonstrated increased oncogenic activity through high C-terminal phosphorylation and dimerization potential [38].

## 3. Epidemiology

De novo *HER2* aberrations are mutually exclusive with other oncogenic driver alterations, such as mutations in *KRAS*, *EGFR*, etc. [25]. They most commonly occur in patients with lung adenocarcinoma; *HER2* mutations and amplifications have been reported in 1–4% and 2–5% of patients with lung adenocarcinoma, respectively [25,32,34,39,40]. Mutations in *HER2* are more frequent in female patients and never-smokers; *HER2* amplification and concomitant protein overexpression have been linked with male sex and cigarette smoking [41,42]. No association with HER2 aberrations and age has been documented. *HER2* amplification also plays a significant role in *EGFR*-mutant NSCLC, emerging as a mechanism of acquired resistance to EGFR TKIs in 10–12% of the cases [43,44,45].

## 4. HER2 Diagnostic Assays

Currently, the two approved techniques for HER2 status evaluation in breast cancer are immunohistochemistry (IHC) and fluorescence in situ hybridization (FISH), which detect HER2 protein overexpression and *HER2* gene amplification (defined as an increased copy number of the chromosome 17 region [17q12q21]), respectively. In brief, according to the updated 2018 American Society of Clinical Oncology (ASCO)/College of American Pathologists (CAP) guidelines, HER2 positivity is defined either as >10% of tumors cells showing complete, strong HER2 membrane staining (3+) by IHC, or as the detection of a *HER2*:*CEP17* ratio of ≥2 and/or *HER2* gene copy number ≥ 6 by FISH. In diagnostically ambiguous cases, where weak to moderate membrane staining is observed in >10% of tumor cells (termed IHC2+), retest of the same specimen with FISH or testing of a new specimen with either IHC or FISH is required [46]. Similar recommendations have been made for gastroesophageal adenocarcinoma, with the exception that due to characteristic histology, the percentage of tumor cells that exhibit basolateral or lateral, rather than complete, membranous staining is calculated to define HER2 positivity by IHC [47]. It should be noted that the previously described cutoffs are closely linked to the clinical indication for trastuzumab administration, as the mechanism of action and consequently the efficacy of the drug is based on HER2 protein overexpression [48].

Regarding NSCLC, no official guidelines have been developed for the assessment of HER2 positivity, and as a result, the evaluation of gene amplification and/or protein overexpression in clinical trials has been, until recently, performed via extrapolation from breast and gastric cancer. Notably, HER2 IHC staining in NSCLC exhibits better analogy with gastric rather than breast cancer staining patterns [49]. Importantly, as far as NSCLC is concerned, “*HER2*-mutant” is becoming a considerably more relevant term than “HER2-positive”. Moreover, *HER2* amplification and/or overexpression have failed to demonstrate any clear or consistent association with the presence of mutations in *HER2* across several studies [25,27,50,51]. Poziotinib recent fast track therapy designation was granted based on the presence of *HER2* exon 20 mutations, which were documented either in tissue by next-generation sequencing (NGS) with Oncomine™ Comprehensive Assay (Thermo Fisher Scientific, Waltham, MA, USA) or FoundationOne^®^ Assay (Foundation Medicine, Cambridge, MA, USA) or in plasma using Guardant360^®^ Assay (Guardant Health, Redwood City, CA, USA) [52]. In the Destiny-Lung01 trial, which led to the breakthrough therapy designation of trastuzumab-deruxtecan for *HER2*-mutant NSCLC, *HER2* mutation status was determined again by a CLIA standards local NGS method or equivalent and then centrally confirmed with the Oncomine™ Dx Target Test (Thermo Fisher Scientific, Waltham, MA, USA). Inclusion criteria were met for patients harboring a known *HER2*-activating mutation of the kinase (exons 18–20), juxtamembrane (exon 17), or extracellular domain (exon 8) [53].

## 5. Targeting HER2 in NSCLC

*Non-selective tyrosine kinase inhibitors.* The first attempts to target *HER2* in patients with NSCLC were made using non-selective TKIs already approved for the treatment of *EGFR*-positive disease. In 2015, De Grève et al. assessed the efficacy of the quinazoline-based, irreversible pan-HER TKI afatinib in pretreated patients with advanced or metastatic NSCLC harboring *HER2* exon 20 mutations (Table 1) [54]. Neither complete (CR) nor partial responses (PR) were reported. However, stable disease (SD) was documented in five out of seven study participants, accounting for a disease control rate (DCR) of 71.4%. Subsequently, afatinib was evaluated as part of a named patient use program that enrolled 28 heavily pretreated patients with advanced NSCLC and activating *HER2* mutations determined by local testing [55]. Among 16 patients with available tumor response data, three PR were documented, conveying an objective response rate (ORR) of 18.8% and DCR of 68.8%. Notably, the efficacy of afatinib was higher in patients harboring the A775_G776insYVMA mutation, with an ORR of 33.3% and DCR of 100%. The NICHE phase II study investigated the potential of afatinib to control disease in patients with *HER2*-mutant disease that had progressed during or after platinum-based chemotherapy [56]. Only one patient achieved PR, while ORR and DCR were 7.7% and 53.8%, respectively, resulting in the accrual to stop at 13 patients. Median progression-free survival (PFS) was 15.9 weeks, and median overall survival (OS) was 56.0 weeks. Lately, the efficacy of afatinib was evaluated in the setting of another phase II trial conducted in previously treated Asian patients with *HER2*-mutant NSCLC [57]. This study consisted of two parts: in Part A, patients were treated with afatinib monotherapy until disease progression or unacceptable toxicity, while Part B were to enroll patients who showed clinical benefit in Part A (for more than 12 weeks) and continue treatment with afatinib coupled with paclitaxel. While DCR was 61.1%, none of the 18 enrolled patients achieved a PR. Median PFS and OS were 2.8 months and 10.0 months, respectively. Due to the lack of study participants meeting the inclusion criteria for Part B as well as the slow rate of recruitment, the study was terminated early. In all the above studies, adverse events (AE) were generally consistent with the toxicity profile of afatinib, with the most common being diarrhea, rash/acne, and stomatitis.

Dacomitinib is another irreversible HER family blocker that has shown efficacy in *EGFR*-mutant NSCLC [58,59]. The antitumor activity of dacomitinib was evaluated in a prespecified cohort of a phase II study, which included patients with advanced or metastatic NSCLC and *HER2* mutations (*n* = 26) or amplification (*n* = 4) [60]. ORR was 11.5% in patients with *HER2* mutations but 0% in those with amplifications. In patients with *HER2*-mutant tumors, the median PFS and OS were 3.0 and 9.0 months, respectively. Interestingly, exon 20 mutations did not correlate with gene amplification. In addition, no responses were observed in the presence of the most common *HER2* exon 20 alteration, namely A775_G776insYVMA. Treatment-related AEs occurred in all study participants; the most common AE was diarrhea, which was documented in 90.0% of the patients. Dacomitinib toxicity led to dose reduction or discontinuation in 17.0% and 13.0% of the patients, respectively.

The in vitro activity of HER-targeted agents across different *HER2* mutations has been evaluated in preclinical NSCLC models, with both afatinib and neratinib exhibiting significantly increased efficacy toward the A775_G776insYVMA exon 20 insertion [61]. Furthermore, the combination of neratinib and trastuzumab was more effective than neratinib monotherapy, resulting in robust inhibition of HER2 as well as downstream signaling in vivo [62]. Twenty-six patients with advanced NSCLC and concomitant *HER2* aberrations received neratinib in the setting of the SUMMIT phase II basket study [63]. Despite the low ORR observed (3.8%, one PR in a case with L755S kinase domain missense mutation), DCR reached 42.3%. The median PFS was 5.5 months, with six patients remaining on treatment for more than a year, suggesting that neratinib may still have a positive impact on the natural course of refractory NSCLC cases. The most common AEs of neratinib were diarrhea (73.8%), nausea (43.3%), and vomiting (41.1%).

*Selective tyrosine kinase inhibitors.* Non-selective pan-HER TKIs have demonstrated moderate antitumor activity, achieving sporadic responses against exon 20 insertions that represent the most common genomic aberration in *HER2*-mutant NSCLC. Three-dimensional modeling revealed that such insertions induce a constitutively active conformation, which prevents the binding of non-covalent HER2 inhibitors. Additionally, exon 20 insertions appear to confine the drug-binding pocket, hampering the binding of large, rigid inhibitors and the size of the insertion may affect drug sensitivity; single amino acid insertions are more likely to respond to, previously mentioned, non-selective TKIs (i.e., afatinib) [64]. In this regard, poziotinib has been shown to possess certain structural features, including relatively small size, increased halogenation, and flexibility, that could overcome insertion-induced changes within the drug-binding pocket of exon 20. Poziotinib had an average IC50 value of 1.9 nM in Ba/F3 cell lines with an *HER2* exon 20 insertion, making it six times more potent than afatinib in vitro. The predicted efficacy of poziotinib was also confirmed in vivo using NSCLC tumor models that bore the previously described genomic alterations. Early results of poziotinib from phase I trials justified its further development in patients with *EGFR*-mutant as well as *HER2*-mutant cancers [65]. Robichaux et al. conducted a phase II study in 12 heavily pretreated NSCLC patients harboring *HER2* exon 20 insertions with promising preliminary results; ORR was 41.7% (5 PR), DCR was 83.3%, and median PFS was 5.6 months [66]. All study participants harbored either the Y772dupYVMA or G778dupGSP insertions. The most common treatment-related AE was rash, which occurred in all study participants and was grade 3 or 4 in seven cases. Other common AEs were diarrhea and paronychia, which were mainly grade 1 or 2. Eight patients on poziotinib (66.7%) required at least one dose reduction, but no patient had to discontinue treatment due to poziotinib-related toxicity. Similar results were reported from another investigator-initiated, single-center study that enrolled 30 NSCLC patients with *HER2* exon 20 mutations [52]. ORR for poziotinib was 26.7% with responses detected across different *HER2* exon 20 mutation subtypes. The median PFS reached 5.5 months, and median OS reached 15 months. Matters of safety and efficacy of poziotinib were also evaluated in ZENITH20, a multi-cohort phase II clinical trial, including both previously treated and treatment naïve patients with *HER2* exon 20 mutations (cohorts 2 and 4, respectively) [67]. In March 2021, the FDA granted “fast-track designation” to poziotinib for use in previously treated patients with HER2 exon 20 mutations based on preliminary results from the ZENITH20 study; ORR was 27.8%, DCR was 70.0% and median PFS was 5.5 months [68]. Updated results from cohort 4 also suggested promising antitumor activity among treatment-naïve patients with an ORR of 43.8%, DCR of 75.0%, and median PFS of 5.6 months [69]. The toxicity profile of poziotinib was consistent with the previous studies leading to dose interruptions, dose reductions, and treatment discontinuation in 88.0%, 76.0%, and 12.0% of patients, respectively. Lately, data from the poziotinib expanded access program came to confirm the phase II results; the ORR was 30.0% (higher in patients with *HER2*-mutant tumors), DCR was 80.0%, median PFS was 5.6 months, and median OS was 9.5 months [70].

However, heterogeneity among *HER2* exon 20 insertions affects sensitivity to anti-HER2 TKIs, and C805S insertion has been shown to mediate acquired drug resistance in initially sensitive *HER2* exon 20 insertion models [71]. Using patient-derived tumor organoids as well as xenografts, Wang et al. demonstrated the enhanced antitumor activity of pyrotinib [72]. Pyrotinib also produced promising preliminary results in 15 previously treated patients with an *HER2*-mutant NSCLC. The ORR was 53.3% (8 PR) and DCR was 73.3% with a median PFS of 6.4 months. Notably, pyrotinib was effective against A775_G776insYVMA, G776C, G776>VC, and L755P but not the G776>IC mutation. Subsequently, pyrotinib was evaluated in a larger phase II trial that enrolled 60 previously treated stage IIIB–IV NSCLC patients with *HER2*-mutant tumors [73]. The ORR and DCR observed were 30.0% and 85.0%, respectively. Importantly, the administration of pyrotinib benefited all different patient subcategories, including patients with brain metastases at baseline and heavily pretreated individuals. The median PFS reached 6.9 months and the median OS reached 14.4 months. As far as safety is concerned, the most common treatment-related AE was diarrhea, as reported in 91.7% of the patients. Other treatment-related toxicities included elevations in serum creatinine and transaminases.

Tarloxotinib is a hypoxia-activated prodrug of an irreversible pan-HER TKI [74]. Although the prodrug has feeble inhibitory activity in oxygenated normal tissues, sparing them from wild-type HER inhibition, it can be converted to the active metabolite (tarloxotinib-E) in the hypoxic tumor microenvironment. Tarloxotinib-E-induced suppression of EGFR, HER2, and HER4 signaling has been shown to impede tumor cell proliferation in vitro, resulting in tumor regression in multiple murine xenograft models. Pharmacokinetic analysis confirmed significantly increased concentrations of tarloxotinib-E in tumor tissue compared with plasma or normal skin. The RAIN-701 phase II trial enrolled patients with advanced NSCLC harboring *HER2* activating mutations (cohort B) [75]. Among nine evaluable patients, two experienced PR and four had SD, accounting for ORR and DCR of 22.2% and 66.7%, respectively. The most common treatment-related AEs were QTc prolongation, rash, and diarrhea occurring in 60.9%, 43.5%, and 21.7%, respectively.

**Table 1:** Tyrosine kinase inhibitors in patients with NSCLC harboring HER2 aberrations. NA, not available; NR, not reached; TKI, tyrosine kinase inhibitor; RT-PCR, reverse transcriptase polymerase chain reaction; NGS, next-generation sequencing; ORR, objective response rate; PFS, progression-free survival; OS, overall survival; AE, adverse event.

Reference	TKI	Study	N	Previous Treatment Type; N (%)	HER2 Positivity Definition (Method)	ORR N (%)	PFS Median (95% CI)	OS Median (95% CI)	All-Grade AEs (%)
De Grève et al. [54]	Afatinib	Phase II, Basket	7	Chemotherapy; 5 (71.4)	Exon 20 mutation (PCR; central)	0/7 (0)	17 weeks (NA)	–	Diarrhea (95.0), rash/acne (80.0), stomatitis (46.0)
Peters et al. [55]	Afatinib	Phase II, Single-arm	28	Systemic therapy; 26 (92.9)	Activating mutation (NA; local)	3/16 (18.8)	–	–	Diarrhea (35.7), skin disorders (28.6), stomatitis (14.3)
Dziadziuszko et al. [56]	Afatinib	Phase II, Single-arm	13	Chemotherapy; 13 (100.0)	Exon 20 mutation (Various; local)	1/13 (7.7)	15.9 weeks (6.0–35.4)	56.0 weeks (16.3- NR)	Diarrhea (NA), skin disorders (NA), stomatitis (NA)
Fan et al. [57]	Afatinib	Phase II, Single-arm	18	Chemotherapy; 18 (100.0)	Exon 19, 20 mutation (RT-PCR; central)	0/18 (0)	2.8 months (1.9–4.6)	10.0 months (8.5–10.1)	Diarrhea (66.7), rash (33.3), stomatitis (11.1)
Kris et al. [60]	Dacomitinib	Phase II, Single-arm	26	Chemotherapy; 18 (100.0)	Exon 20 mutation (NGS; central)	3/26 (11.5)	3 months (2.0–4.0)	9 months (7.0–21.0)	Diarrhea (90.0), rash (73.0), fatigue (57.0)
Hyman et al. [63]	Neratinib	Phase II, Single-arm	26	Systemic therapy; 26 (100.0)	Activating mutation (NGS; local)	1/26 (3.8)	5.5 months (NA)	–	Diarrhea (73.8), nausea (43.3), vomiting (41.1)
Robichaux et al. [66]	Poziotinib	Phase II, Single-arm	12	Systemic therapy; 11 (91.7)	Exon 20 mutation (NA; NA)	5/12 (41.7)	5.6 months (NA)	–	Rash (100.0), diarrhea (91.7), paronychia (91.7)
Elamin et al. [52]	Poziotinib	Phase II, Single-arm	30	Systemic therapy; 28 (93.0)	Exon 20 mutation (NGS; local)	8/30 (26.7)	5.5 months (4.0–7.0)	15.0 months (9.0-NR)	Skin rash (83.0), Diarrhea (80.0) Paronychia (70.0)
Cornelissen et al. [69]	Poziotinib	Phase II, Randomized	48	No; 0 (0)	Exon 20 mutation (NGS; local)	21/48 (43.8)	5.6 months (NA)	–	NA
Wang et al. [72]	Pyrotinib	Phase II, Single-arm	15	Systemic therapy; 15 (100.0)	Exon 20 mutation (NGS; local)	8/15 (53.3)	6.4 months (1.6–11.2)	–	Diarrhea (26.7), anemia (26.7), hypocalcemia (26.7)
Zhou et al. [73]	Pyrotinib	Phase II, Single-arm	60	Chemotherapy 60 (100.0)	Exon 19, 20 mutation (RT-PCR; central)	18/60 (30.0)	6.9 months (5.5–8.3)	14.4 months (12.3–21.3)	Diarrhea (91.7), elevated creatinine (30.0), vomiting (28.3)
Liu et al. [75]	Tarloxitinb	Phase II, Basket	11	Chemotherapy 11 (100.0)	Activating mutation (NA; local)	2/9 (22.2)	–	–	QTc prolongation (60.9), rash (43.5), diarrhea (21.7)

Mobocertinib is a novel small-molecule TKI designed against exon 20 insertions; in a phase I/II clinical trial, mobocertinib showed promising antitumor activity in previously treated NSCLC patients with *EGFR* exon 20 insertions, resulting in “breakthrough therapy designation” by the U.S. Food and Drug Administration (FDA) [76,77]. Preclinical data indicate that mobocertinib is highly selective, as it possesses the lowest *HER2* exon 20 insertion/wild-type *EGFR* IC50 ratio. In addition, tumors harboring the G776>VC insertion exhibited dramatic and more prolonged responses compared with YVMA insertion-positive tumors. In addition, mobocertinib appeared to act synergistically with T-DM1 in YVMA insertion-positive lung cancer.

*Monoclonal antibodies.* Trastuzumab is a humanized IgG1 kappa mAb that binds to the extracellular domain of HER2. It interferes with HER2 signaling via several mechanisms, including inhibition of receptor dimerization, prevention of extracellular domain shedding, endocytotic destruction of the receptor, and antibody-dependent cell-mediated cytotoxicity [78]. Based on the paradigm of breast cancer, most clinical trials evaluating trastuzumab in patients with NSCLC have required evidence of protein overexpression or gene amplification rather than the detection of specific *HER2* gene mutations. HOT1303-B trial assessed trastuzumab monotherapy in 10 previously treated patients with NSCLC and HER2-altered tumors, which were defined as IHC3+, IHC 2+/FISH+, and/or by the presence of activating mutations (Table 2) [79]. Although no responses were documented, DCR was 70.0%, and the median PFS reached 5.2 months. A larger, randomized phase II trial in 101 treatment-naïve patients with NSCLC, showed that the addition of trastuzumab to cisplatin and gemcitabine was beneficial in a small subgroup of IHC3+ or FISH+ cases (six patients; ORR of 83.3% and median PFS of 8.5 months) but not in the overall population [80]. Similar results were observed in another phase II single-arm study evaluating the combination of paclitaxel, carboplatin, and trastuzumab; ORR was 24.5%, median PFS was 3.3 months, and median OS was 10.1 months [81]. Available data from the MyPathway phase II basket trial demonstrated that dual HER2 blockade with trastuzumab and pertuzumab was effective in patients harboring *HER2* exon 20 mutations as well as those having HER2 amplification or overexpression (ORR was 21.4% and 15.4%, respectively; DCR was 46.2% and 25.0%, respectively) [82].

*Antibody–drug conjugates.* Trastuzumab-emtansine (T-DM1) is an ADC that consists of an anti-HER2 agent (trastuzumab) and a cytotoxic microtubule (emtansine; DM1). T-DM1 employs receptor-mediated endocytosis to enter HER2-positive cells; DM1 is released after proteolytic degradation of the antibody moiety in the target cell lysosomes [83]. The first results of T-DM1 in NSCLC were derived from a small phase II trial that enrolled 15 previously treated patients [84]. T-DM1 demonstrated minimal antitumor activity with an ORR of 6.7%, DCR of 71.4%, and median PFS and OS times of 2.0 months and 10.9 months, respectively. Notably, no responses were obtained in patients with *HER2*-amplified or HER2-overexpressing tumors. However, results from a subsequent phase II basket trial enrolling 49 previously treated patients (including 11 patients with *HER2* amplification and 28 patients with *HER2* mutations) revealed that treatment with T-DM1 might achieve ORR as high as 51.0% [85,86]. Median PFS for the study cohort was 5.0 months. Although study data indicated that the presence of *HER2* mutations detected by NGS was not concordant with gene amplification or protein overexpression by IHC and FISH, responses to T-DM1 were comparable between these subgroups. Peters et al. assessed the efficacy of T-DM1 in 49 previously treated NSCLC patients with HER2 overexpression (i.e., IHC2+ or IHC3+) [87]. Although no responses were seen in the IHC2+ cohort, ORR was 20.0% in the IHC3+ cohort; DCR was 27.6% and 40.0% in the IHC2+ and IHC3+ cohorts, respectively. Median PFS and OS were comparable in the IHC 2+ and 3+ cohorts (median PFS was 2.6 months and 2.7 months, respectively and median OS was 12.2 months and 15.3 months, respectively). The toxicity profile of T-DM1 was consistent with previous trials in patients with breast cancer.

Trastuzumab–deruxtecan (T-Dxd) is a newer HER2-targeting ADC composed of trastuzumab, which is an enzymatically cleavable peptide linker and a topoisomerase I inhibitor (MAAA-1181). T-Dxd is a stable and homogeneous molecule with increased membrane permeability and a higher drug-to-antibody ratio compared with other available ADCs. This enables a steady delivery of the topoisomerase I inhibitor even in HER2-low expressing conditions [88]. The first results of T-Dxd in heavily pretreated (median of four prior anticancer regimens) patients with NSCLC were impressive; ORR was 55.6% (6/11 PR), and DCR was 77.8% with a median PFS of 11.3 months [89]. Patients with *HER2*-mutant non-small cell lung tumors had more pronounced treatment benefit than those without documented mutations, regardless of IHC/FISH status; among 11 patients harboring the *HER2* mutation, the ORR and DCR were 72.7% and 90.9%, respectively. The DESTINY-Lung01 phase II trial evaluated the efficacy of T-Dxd in two cohorts of NSCLC patients; cohort 1 contained patients with HER2 overexpression (i.e., IHC2+ or IHC3+), while cohort 2 contained patients with *HER2* mutations. Results from cohort 1 showed an ORR of 24.5% with a median PFS of 5.4 months. Notably, response rates did not differ according to HER2 IHC expression levels. Recently published results from cohort 2 indicated enhanced antitumor efficacy among patients with *HER2*-mutant tumors [53]. Among 91 patients, the ORR reached 54.9% with a DCR of 92.3%. The median PFS and OS were 8.2 months and 17.8 months, respectively. The most common treatment-related AEs were gastrointestinal and hematologic, while pneumonitis was reported in about 12.0% of the study participants. According to these data, trastuzumab-deruxtecan received “breakthrough therapy designation” from the FDA for the treatment of patients with metastatic NSCLC whose tumors have a *HER2* mutation and with disease progression on or after platinum-based therapy.

**Table 2:** Monoclonal antibodies in patients with NSCLC harboring HER2 aberrations. NA; not available; NR, not reached; IHC, immunohistochemistry; FISH, fluorescence in situ hybridization; ELISA, enzyme-linked immunosorbent assay; NGS, next-generation sequencing; ORR, objective response rate; PFS, progression-free survival; OS, overall survival; AE, adverse event.

Reference	Agent	Study	N	Previous Treatment Type; N (%)	HER2 Positivity Definition (Method)	ORR N (%)	PFS Median (95% CI)	OS Median (95% CI)	All-Grade AEs (%)
Kinoshita et al. [79]	Trastuzumab	Phase II, Single-arm	10	Yes, systemic therapy; 10 (100.0)	Overexpression/ amplification (IHC3+, IHC2+/FISH+; NA), activating mutation (NA; NA)	0/10 (0)	5.2 months (1.4–6.3)	–	–
Gatzemeier et al. [80]	Cisplatin, gemcitabine ± trastuzumab	Phase II, Randomized	101 (50/101 cisplatin, gemcitabine and trastuzumab; 51/101 cisplatin, gemcitabine)	No; 0 (0)	Overexpression/ amplification (IHC2-3+, FISH+, ELISA+; local)	18/50 (36.0); 21/51 (41.2)	6.1 months (0.1–19.6); 7.0 months (6.0–7.7)	12.2 months (0.1–19.6); NR	Nausea (74.0), vomiting (46.0), fatigue (42.0)
Langer et al. [81]	Carboplatin, paclitaxel, trastuzumab	Phase II, Single-arm	53	No; 0 (0)	Overexpression/ amplification (IHC1-3+; local)	13/53 (24.5)	3.3 months (NA)	10.1 months (6.7–14.6)	Anemia (99.0), fatigue (71.0), sensory neuropathy (65.0)
Hainsworth et al. [82]	Trastuzumab, pertuzumab	Phase II, Basket	30 (16/30 HER2 overexpression/ amplification; 14/30 *HER2* mutation)	Yes, systemic therapy; NA	Overexpression/ amplification (IHC3+, FISH+; local); exon 20 mutation (NGS; local)	5/30 (16.7) [2/13 (15.4); 3/14 (21.4)]	–	–	–
Hotta et al. [84]	T-DM1	Phase II, Single-arm	15	Yes, systemic therapy; 15 (100.0)	Overexpression/ amplification (IHC3+, IHC2+/FISH+; central), exon 20 mutation (NGS; central)	1/15 (6.7)	2 months (1.4–4.0)	10.9 months (4.4–12.0)	–
Li et al. [85]	T-DM1	Phase II, Single-arm	49 (11/49 *HER2* amplification; 28/49 *HER2* mutation)	Yes, systemic therapy; 49 (100.0)	Activating mutation (NGS; local)	25/49 (51.0) [6/11 (54.5); 14/28 (50.0)]	5 months (3.5–5.9)	–	Elevated LFTs (63.3), thrombocytopenia (30.6), nausea (28.6)
Peters et al. [87]	T-DM1	Phase II, Single-arm	49 (29/49 HER2 IHC2+; 20/49 HER2 IHC3+)	Yes, systemic therapy; 49 (100.0)	Overexpression (IHC3+, IHC2+; central)	4/49 (8.2) [0/29 (0); 4/20 (20.0)]	2.6 months (1.4–2.8)	12.2 (4.7–23.6)	Infusion reaction (14.3), peripheral neuropathy (14.3), hemorrhage (14.3)
Tsurutani et al. [89]	T-Dxd	Phase I, Single-arm	18	Yes, systemic therapy; 18 (100.0)	Overexpression/ amplification (IHC1+, FISH+, NGS; local)	10/18 (55.6)	11.3 months (5.5–14.1)	17.3 months (17.3-NR)	Nausea (74.6), vomiting (52.6), anemia (39)
Li et al. [53]	T-Dxd	Phase II, Two arms	49 HER2 overexpression; 91 *HER2* mutations	Yes, systemic therapy; 140 (100.0)	Activating mutation (NGS; local)	12/49 (24.5); 50/91 (54.9)	5.4 months (2.8–7.0); 8.2 months (6.0–11.9)	NA; 17.8 months (13.8–22.1)	Nausea (73.0), fatigue (53.0), alopecia (46.0)

*Immune checkpoint blockade.* The presence of driver alterations in NSCLC has been associated with a “cold” immune microenvironment and the limited clinical efficacy of immune checkpoint inhibitors [90,91,92]. Among different driver alterations, mutations in *HER2* have been linked with the lowest levels of PD-L1 expression, which are comparable to those of tumors harboring either classic or exon 20 *EGFR* mutations; the prevalence of positive and high PD-L1 expression in *HER2*-mutant disease was about 50.0% and 20.0%, respectively. Accordingly, NSCLC tumors with *HER2* mutations have in general low tumor mutational burden (TMB), with median TMB values < 3 mut/Mb.

As far as efficacy is concerned, available data are scarce and extracted solely from retrospective studies (Table 3). NSCLC patients with *HER2*-mutant tumors that receive immunotherapy beyond the first line of treatment generally exhibit single-digit ORRs [93,94,95,96,97]. For such patients, median PFS and OS fall within the range of 2 months and 20 months, respectively. Recently, Saalfeld et al. reported on the efficacy of immunotherapy (either alone or combined with chemotherapy) in patients with *HER2*-mutant NSCLC comparing patients in the first versus subsequent lines of therapy [98]. Using a retrospective cohort of 61 patients, they found that first-line chemoimmunotherapy may achieve response rates comparable to those of unselected NSCLC patients; among patients receiving first-line chemoimmunotherapy, the ORR was 52.4% and the median PFS reached 6.0 months. However, results were inferior for patients treated with programmed cell death protein 1 (PD-1)/programmed death-ligand 1 (PD-L1) axis inhibitors in the second or subsequent line of therapy.

## 6. Conclusions

As new drug designations rapidly alter the treatment landscape of *HER2*-mutant NSCLC, several matters still need to be addressed. First, mature OS data as well as phase III trials are expected to confirm the therapeutic benefit from such therapies and compare them with the current standard of care. Second, in an effort to define *HER2*-mutant disease and determine patterns of response among different *HER2* variants, it is imperative that future guidelines advocate for standardized assays for the detection of activating *HER2* mutations. In addition, the propensity of *HER2*-mutant NSCLC for central nervous system (CNS) involvement during the course of treatment will require tailor-made algorithms for the early identification of CNS metastases as well as careful assessment of the intracranial activity of the existing and upcoming anti-HER2 agents. Finally, definitive formulation of the relationship between HER2 amplification/overexpression and activating mutations is urgently needed, since it will inform treatment decisions, including in patients with acquired resistance to EGFR TKIs.

## Figures and Tables

**Table 3:** Immune checkpoint inhibitors in patients with NSCLC harboring HER2 aberrations. NA; not available; NR, not reached; ICI, immune checkpoint inhibitor; PD-L1, programmed death-ligand 1; ORR, objective response rate; PFS, progression-free survival; OS, overall survival; AE, adverse event.

Reference	Study	N	Line of Treatment, ICI Regimen	PD-L1 Expression ≥1/≥50 (%)	ORR N (%)	PFS Median (95% CI)	OS Median (95% CI)
Lai et al. [93]	Retrospective	26	NA	NA/8.7	3/26 (11.5)	1.9 months (1.5–4.0)	10.4 months (5.9-NR)
Negrao et al. [94]	Retrospective	16	NA	NA/NA	1/16 (6.3)	1.8 months (NA)	17.1 months (NA)
Mazieres et al. [95]	Retrospective	29	>1, monotherapy	53.3/0	2/27 (7.4)	2.5 months (1.8–3.5)	20.3 months (7.8-NR)
Guisier et al. [96]	Retrospective	23	>1, monotherapy	17.4/4.3	6/23 (27.3)	2.2 months (1.7–15.2)	20.4 months (9.3-NR)
Lau et al. [97]	Retrospective	14	>1, monotherapy	61.5/23.1	4/14 (28.6)	3.6 months (1.6-NR)	NA
Saalfeld et al. [98]	Retrospective	61	1, monotherapy (5/61); 1, combination with chemotherapy (22/61); >1, monotherapy (34/61)	53.4/15.5	1/5 (20.0); 11/21 (52.4); 5/31 (16.1)	NA; 6 months (6.0–14.0); 4 months (4.0–6.0)	NA; NR; 10 months (6.0-NR)

## Data Availability

Data sharing not applicable.
